# Return to sports after ACL injury 5 years from now: 10 things we must do

**DOI:** 10.1186/s40634-022-00514-7

**Published:** 2022-07-30

**Authors:** Alli Gokeler, Alberto Grassi, Roy Hoogeslag, Albert van Houten, Tim Lehman, Caroline Bolling, Matthew Buckthorpe, Grant Norte, Anne Benjaminse, Pieter Heuvelmans, Stefano Di Paolo, Igor Tak, Francesco Della Villa

**Affiliations:** 1 Centre for Orthopaedic Surgery and Sports Medicine OCON, Hengelo, The Netherlands; 2 Department of Public and Occupational Health, Amsterdam Movement Sciences, Amsterdam Collaboration On Health and Safety in Sports, Amsterdam UMC, Amsterdam, Netherlands; 3 Department Exercise and Health, Faculty of Science, Exercise Science and Neuroscience, Paderborn University, Paderborn, Germany; 4 IRCCS Istituto Ortopedico Rizzoli, Bologna, Italy; 5 Allied Health and Performance Science, St Mary’s University, Twickenham, London England; 6 Exercise Science Program, School of Exercise and Rehabilitation Sciences, University of Toledo, Toledo, USA; 7 Center for Human Movement Sciences, University of Groningen, University Medical Center Groningen, Groningen, Netherlands; 8 School of Sport Studies, Hanze University Groningen, Groningen, the Netherlands; 9 Dipartimento Di Scienze Biomediche E Neuromotorie DIBINEM, Università Di Bologna, Bologna, BO Italy; 10 Sports Physical, Therapy Clinic Fysiotherapie Utrecht Oost, Utrecht, The Netherlands; 11 Education and Research Department, Isokinetic Medical Group, FIFA Medical Center of Excellence, Bologna, Italy

**Keywords:** ACL, Surgery, Strength, Neuroplasticity, AMI, Biomechanics, Psychological, Context, Return to sports

## Abstract

**Background:**

The outcome after ACL reconstruction (ACLR) is in general disappointing with unacceptable number of athletes that do not return to pre-injury level of sports, high re-injury rates, early development of osteoarthritis and shorter careers. Athletes after ACLR have high expectation to return to sports which is in contrast with the current outcomes. The aim of this manuscript is to present an overview of factors that are needed to be incorporated and to personalize the rehabilitation process for an athlete who has undergone an ACLR.

**Level of evidence:**

4.

## Key Points

1. A tailored surgical procedure is needed based on the patient’s desired sport activity, anatomical features, laxity pattern and concomitant injuries.
2. A growing evidence base supports the use of neuromodulatory interventions to address the underlying pathophysiology contributing to muscular impairments following ACLR.
3. Compensatory movements should be targeted with motor learning principles in the early phase of ACLR rehabilitation to optimize outcome.
4. Clinicians should create a rich rehabilitation environment that includes sensory and cognitive stimulation adjuvant to motor tasks.
5. Clinicians should recognize that psychological, social, and contextual factors are critical factors for successful recovery after ACLR.
6. Athletes after ACLR should be prepared for the physical demands of their sport and include sport-specific physical performance testing
7. Wearable sensor technology should be used during on-field sport-specific situations to assess movements and performance.
8. The future questions for rehabilitation and RTS should focus not only on which criteria or what works -we need to develop new questions about what works for which context, for whom, and when some criteria are relevant.

## Introduction

Athletes who sustained an injury of the anterior cruciate ligament (ACL) have high expectations (88%) after subsequent ACL reconstruction (ACLR) to return to pre-injury level of sports [42]. However, only 55% of patients after ACLR return to the competitive level of sport [5].

The competitive, young athlete (< 20 years) who resumes pivoting type sports following ACLR has a high risk for second ispi- or contralateral ACL injury. Injury rates in this young cohort have been reported of up to 29–40% in the literature [94, 128, 130].

Consequently, there is growing interest in validating RTS criteria aiming to reduce the rate of a second ACL injury. Despite the development of return to sport (RTS) guidelines over recent years, there is an ongoing debate pertaining the validity of these RTS criteria [129]. Typically, these are a set of criteria or “test battery” that is used to clear the athlete for RTS at the final stage of rehabilitation [36]. The decision as to when an athlete is allowed to RTS however, is multifactorial, difficult, and challenging [138]. An essential quartet for recovery has been proposed: 1) correct diagnosis, 2) restoration of anatomy, 3) biological healing, and 4) functional rehabilitation [106]. Although it goes without saying that proper medical care is fundamental and that time for the graft to mature is needed for recovery, the same holds true for the quality of rehabilitation. It is apparent that current rehabilitation programs do not yet effectively target strength deficits [119], postural stability [69] and aberrant movement patterns after ACLR [47, 64, 102]. Quadriceps weakness and aberrant movement patterns are suggested to be risk factors for an early onset of osteoarthritis and second ACL injury risk [74, 88].

Components of current rehabilitation programs entail a combination of exercises to increase muscle strength and endurance and improve neuromuscular function [115]. Although we acknowledge the importance of addressing these factors, there is a clear need for improvement.

It has been recognized that an ACL injury may induce neurophysiological alterations affecting restoration of function, however this knowledge has yet to be implemented by clinicians in the rehabilitation programs [83, 87, 108].

Another underrepresented domain in rehabilitation are neurocognitive factors which are linked to initial ACL injury risk factors [48, 56, 118]. The current RTS functional tests are relatively simple motor tasks performed in a predictable environment and as such do not reflect the complex demands an athlete faces upon return to the field.

A more holistic approach to an injured athlete is thus needed. Specifically, we must acknowledge that there is a human being attached to the injured knee. The knee is thus just one piece of the puzzle of a complex biological system with different biopsychosocial components. Psychological factors have received increased interest over the last decade [6]. Clinicians need to be cognizant of social, contextual and psychological factors and how these influence rehabilitation [121]. Athletes may respond differently to the same type of surgery and rehabilitation strategies. Karlsson and Becker [62] called for an individualized approach to better understand the injured athlete, the specific requirements and the demands as well as the athlete’s wishes. Is that a return to sports, despite the inherent risk of a re-injury? Are patients well enough educated about the long-term risks of developing osteoarthritis? Many questions arise.

The aim of this manuscript is to present an overview of factors that are needed to be incorporated, to optimize and personalize the rehabilitation process for amateur as well as professional athletes who have undergone an ACLR. For both levels of sports, an ACLR results in significant challenges to achieve pre-injury level of sports [5, 85], heightened risk for second ACL injury [124, 128] and shorter careers [7, 85].

## Surgical advancements and considerations

Knee surgeons have always had great interest in ACL injuries, facilitating advancements and innovations in ACL surgery at a high rate uncommon in orthopedic surgery [51]. Thus, indications and techniques for ACLR change year after year following new evidence from biomechanical studies, after the release of new devices and also following the introduction of cutting-edge “theories” [43, 113]. A clear and paradigmatic example is how the scientific community reacted to the discovery of a “new ligament in the knee” [30]: the so-called “Antero-Lateral Ligament”. Since then, hundreds of studies have been published trying to shed light on its anatomy, biomechanics and the effects of its reconstruction [75, 137]. This almost 10-year long process culminated with a multicenter randomized study that proved that adding an extra-articular tenodesis to ACLR in fact decreases the risk of failure in high-risk patients [44]. Thus, the role of anterolateral structures and its surgical management represents the present; but is not considered to be a novelty for the future as in the here and now it should be considered as a standard approach! However, this process represents a trend that is becoming consolidated in the clinical practice of knee surgery: isolated ACLR is becoming increasingly uncommon, especially when dealing with high-demand (e.g., work) patients and athletes. There is also an increased awareness of the importance of the menisci. If the “save the meniscus” mantra [81, 100] is pursued and applied rigorously, more and more meniscal lesions, once considered not amenable to repair, will be sutured, with the hope to preserve joint cartilage and prevent osteoarthritis.

Other emerging trends pertain to the detrimental role of subtle laxities, such as those due to chronic MCL injuries [1, 117], and the stress-increasing effect on the ACL of a steep posterior tibial slope [12, 52, 127]. Thus, in the very near future there will be an increase of ancillary procedures to the ACLR, such as MCL repair or reconstruction, cartilage repair, meniscus repair and slope-correcting high tibial osteotomy. Conversely, a poor performed surgery, defined as the lack to address concomitant injuries and the failure to preserve the menisci will result in suboptimal results of ACLR in most cases, hindering the rehabilitation process and jeopardizing safe and effective return to play outcomes [44, 82, 117].

Taken this all together illustrates that the “isolated ACL reconstruction” is likely becoming a reserved surgery for a small group of cases. In the next 5 years we expect ground-breaking discoveries and technological revolutions. Building on the present standard where concomitant meniscal, ligamentous, and osseous procedures will become the rule (standard of practice) rather than the exception.

This could produce a shift of the ACL injuries management from a standard approach to a more individualized approach where for each single patient, a tailored surgical procedure is performed based on the patient’s desired sport activity, anatomical features, laxity pattern and concomitant injuries. From the perspective of “preservation first”, ACL repair instead of ACL reconstruction might even make a comeback as well [57, 58] and recovery of (some of the) sensory information and lack of donor site morbidity might assist the rehabilitation after ACL repair surgery.

What will this mean for rehabilitation in the next 5 years? Undoubtedly, the scenario’s will become more diverse and complex, since graft selection, fixation methods and patients' sports activity will not be the only factors to consider. Rehabilitation specialists need to be cognizant of the protection of cartilage and meniscal repairs (e.g. complete redial tear, ramp lesion or root repair), combat the risk of stiffness of the multi-ligament reconstruction and take osseous site healing into consideration. In line with the individual surgical approach, the rehabilitation needs to be individualized as well.

## Neurophysiological effects of ACL injury: treating arthrogenic muscle inhibition

Once believed to simply reflect a local musculoskeletal injury, we now understand joint trauma to result in a complex neurophysiological response. In the case of ACL injury, more than two decades of literature suggests that widespread, systemic adaptations occur throughout the nervous system, which are theorized to impede muscular recovery [71]. Following injury and during early recovery from ACLR, a disruption of joint homeostasis (e.g., effusion, pain, inflammation, laxity) changes the transmission of neural signalling from joint mechanoreceptors to the central nervous system, commonly manifesting as quadriceps weakness, activation failure, and atrophy [95]. This characteristic phenomenon, in which uninjured muscle becomes reflexively inhibited due to injury of the joint it surrounds, is termed arthrogenic muscle inhibition (AMI) [59] Understanding the arthrogenic response provides an opportunity for novel intervention strategies to promote quadriceps recovery following ACLR [87].

### Strategy 1: Remove the inhibition (“open and exploit”)

Previous authors [54] have advocated for an “open and exploit” strategy, in which the inhibition is first removed (opened motor neuron pool) then treated with exercise during a therapeutic window to maximize the benefits of rehabilitation (exploited). *Transcutaneous electrical nerve stimulation* (TENS), *focal joint cooling*, and *vibration* (whole-body and local) have been employed to leverage this strategy among those with knee injuries by altering the sensory response from the injured joint [87]. High-frequency sensory TENS applied to the anterior knee before and during exercise has improved quadriceps central activation and strength over a 45-min period and following 2 weeks of use [53]. Similarly, cryotherapy applied to the knee for 20 min prior to exercise has yielded similar benefits [53, 54]. Once the patient is fully weight-bearing and capable of performing prolonged muscle contractions, single [91] and repeated bouts [114] of vibration therapy before and during exercise have increased quadriceps muscle activity, central activation, and strength. Based on the available evidence, the sensory distribution of a joint appears to be an important factor in treating AMI.

### Strategy 2: Divert resources (“send help”)

Quadriceps AMI is largely reflexive, thus mediated by inhibition of motor neurons within the spinal cord. Therefore, increasing neural signalling to skeletal muscle by diverting cortical resources may be advantageous to overcome the inhibition. *Eccentric cross-exercise* and *biofeedback* leverage this “send help” strategy and are well suited to enhance muscle function during early recovery from ACLR [87]. Eight weeks of eccentric cross-exercise has facilitated spinal-reflexive and corticospinal excitability, [73] as well as strength [93] of the non-exercised quadriceps. Likewise, single [14, 96] and repeated [70] bouts of electromyographic- or force-based visual biofeedback have improved quadriceps strength and corticospinal excitability, presumably by enhancing motor neuron recruitment and rate of discharge. While cortical drive to the quadriceps is lower following ACLR, [107] the hamstrings are uniquely facilitated [110], which may further inhibit the quadriceps via reciprocal inhibition. In this way, a single bout of *hamstrings fatiguing exercise* has been used to decrease antagonist-agonist coactivation, while increasing quadriceps central activation [135]. Therefore, diverting cortical resources may aid in optimizing quadriceps function.

### Strategy 3: Circumvent the inhibition (“damage control”)

Minimizing quadriceps atrophy is a common clinical priority following ACLR, yet disruption to neural signalling from the injured joint and integration throughout the nervous system pose inherent challenges. Thus, circumventing the inhibition may present an opportunity to preserve muscle function in the presence of inhibition. *Neuromuscular electrical stimulation* (NMES) and *blood flow restriction* (BFR) can be used in conjunction with therapeutic exercise to leverage this “damage control” strategy [87]. NMES is widely used following ACLR to bypass inhibited motor neurons by stimulation using the motor nerves of skeletal muscle directly. Four to 12 weeks of NMES initiated after the first post-surgical week has improved quadriceps strength, while minimizing atrophy [55, 120].Given that high-load strength training is not possible during this time, BFR can be used to maximize the benefits of low-load exercise by inducing a release of hypertrophic growth factors. One to six sessions of BFR per week over the first day to 16 weeks after surgery have improved quadriceps strength and cross-sectional area [77]. Accordingly, therapeutic adjuncts capable of minimizing tissue damage are well suited to preserve quadriceps function in the presence of inhibition.

### Implementation

More than two decades of literature support intervention strategies to overcome AMI, [87] yet recent work [108] suggests a large constituency of clinicians do not utilize them in practice and perceive several barriers to their implementation (e.g., difficulty quantifying and a lack of formal education on AMI). Future work must attempt to address these barriers and improve the translation of this work to rehabilitation clinicians to effectively advance clinical practice.

## Consider neurophysiological effects of ACL injury II: neuroplasticity for movement

Evidence is emerging that neural adaptations are associated with aberrant motor control of the knee following ACL injury [84]. Alterations in sensory information may go along with decreased innervation to the primary sensory cortex [123], corticospinal and motor cortex excitability [15, 72, 97] in patients after ACLR. As a consequence, greater transcortical stimulation is required to evoke efferent neural signaling in the motor cortex to control movement of the knee joint [72]. Thus, neuroplastic adaptations in different areas of the brain may facilitate the restoration of knee motor control and stability in ACL patients by e.g. driving compensatory synergistic muscle patterns [31].

In this context, athletes after ACLR, may require higher involvement of neurocognitive resources in the frontal cortex for precise joint positioning or lower limb force control [9, 10]. Moreover, high activations involving parieto-occipital cortical areas associated with spatial cognition and orientation, as well as visual-motor processing, have been found linked to motor control during functional motor tasks [32, 68]. Unfortunately, sensorimotor control of the injured lower extremity may appear to rely on visual information processing and cortical motor planning [89]. This, in turn, may limit the individual’s capacity to manage complex motor situations and subsequently predispose ACLR patients to recurrent injury after their return-to-sports [83, 98].

### Implications for rehabilitation

Current rehabilitation programs may not effectively target aberrant movement patterns after ACLR [50, 98]. In light of the aforementioned CNS changes, Gokeler et al. [49] posited that rehabilitation in patients after ACL injury should ideally include sensory and cognitive variations in order to reduce dependency on visual information and in turn facilitate the ability of the brain to achieve novel strategies to cope with altered afferent information from the knee joint. Clinicians should provide a rich enrichment that promotes neuroplasticity throughout various brain regions [86]. Enrichment-induced stimulation of neuronal and synaptic connectivity provides a mechanism for how the brain may utilize existing neuronal networks more efficiently and recruit alternative networks when required [86]. Specifically, enhanced sensory stimulation including perturbations in somatosensory and/or visual input, as well as additional cognitive load, may help to design individually tailored rehabilitation programs. The variability of movement execution in these different conditions may thus be key to effective motor (re-) learning [50] and conducive neuroplastic changes, which finally shape adaptive motor behavior beyond physical rehabilitation [33, 105].

Therefore, it is paramount to consider variability and diversity of sensory and neurocognitive stimulation in ACL rehabilitation programs, to provide patients with a wide variety of motor strategies for adequately solving diverse situations. However, individual responsiveness to corresponding exercise programs, as well as compensatory neuroplastic adaptations still need further exploration.

## Psychological, social and contextual factors after an ACL injury

Clinicians should recognize that psychological, social, and contextual factors are critical factors for successful recovery. It is very likely for an athlete to experience negative emotions at some point after the ACL injury, which hinders recovery [26]. Self-efficacy, self-motivation, fear of reinjury, avoidance behavior and rehabilitation adherence, and support have emerged as important factors for rehabilitation compliance, return to sport, and self-rated knee symptoms [133]. Clearly, what the problem is has been identified quite well, however, less is known about how to address this.

### Implications for rehabilitation—psychological

Responses commonly seen after ACL injury are avoidance behavior and rehabilitation adherence [26]. This behavior is greatly influenced by both cognitive and affective responses [26]. Both will be explained below.

### Self-efficacy

Self-efficacy is an individual’s situation-specific confidence about task outcomes [8]. Higher levels of self-efficacy, or one’s own belief in capabilities during rehabilitation, predict improved knee symptoms, function, physical activity after ACLR and likelihood of RTS [29, 133]. How does this work? In rehabilitation, self-efficacy expectations during exercises are important for the athlete. High confidence has been linked to lower perceived disability [20] and is a predictor of performance [41].

How to increase confidence? It is advised to shape rehabilitation in such a way that athletes’ feelings of perceived disability are decreased and performance expectancies are enhanced. First, this can be done by making sure the athlete receives positive feedback. Providing feedback mainly after good instead of bad trials results in more effective learning. A positive feeling about the task at hand improves goal-action coupling and creates a focus on the task goal and reduces self-focus.

Moreover, adherence to rehabilitation is enhanced with rehabilitation being enjoyable and challenging [99]. An environment that promotes autonomy-supported behavior is associated with greater levels of adherence to rehabilitation and motivation [28]. Athletes value to have an active role in their recovery, be engaged in decision-making, and have their autonomy respected [122]. The clinician is responsible for the exercise program; however, it is advised to give the athlete some control over e.g. order of exercises and number of repetitions [112].

### Implications for rehabilitation—social

It is recommended to train athletes together with peers, without losing individual attention, to provide an environment where athletes feel supported in sharing their experiences and feelings [131]. Social support and engagement in care were the two themes identified in the social domain. The needs for social support change over time and continued re-evaluation of these needs are required [121].

### Implications for rehabilitation—context

Having a strong support system both in and out the rehabilitation setting is a key factor in building a patient’s confidence [132]. An individual’s degree of social support is believed to modulate the psychological stress which comes with the ACL injury, surgery and long rehabilitation period. Perceived social support also appears to affect objective outcomes such as rates of RTS [67].

Especially younger athletes (< 30 years) may benefit from social support from their teammates and coaches, supporting their athletic identity. Family, friends but also peer-patients play an important role in support. Social support within these groups of patients is associated with a positive recovery trajectory following ACLR [37] and mitigate RTS anxiety [109].

## Improve physical conditioning

The RTS outcomes following ACLR, as discussed, are unsatisfactory. In relation to this section, many athletes across different sports return to sport at lower performance levels [85]. It is likely that most rehabilitation approaches are not comprehensive enough, do not provide sufficient intensity or are not specific enough to fully prepare an athlete for the demands of their sport [22].

Successful RTS should involve a continuum from rehabilitation to performance [22]. It is not only about resolving impairments at the knee, but also restoring neuromuscular performance (e.g., maximal strength, power, rate of force development and reactive strength), sports-specific movement quality and sport-specific readiness (fitness, technical training, and chronic training loads) [22, 24, 25]]. To achieve this, we need to think about ‘return to performance’ throughout the functional recovery process [4, 22], but particularly towards the later stages. The rehabilitation and RTS process after ACLR is generally long (~ 6–12 months depending on the sport and level, which can offer an opportunity to develop an athlete’s physical fitness to higher levels than before the injury, as long as specific reconditioning is appropriately incorporated.

Reconditioning can be defined as ‘*re-establishing and/or improving an athlete ‘s overall physical fitness after an injury or surgery*’ and is the author's belief should fit alongside the standard ‘rehabilitation’ model. Whilst fitness reconditioning is a larger priority during the later stages (e.g., late-stage rehabilitation and RTS training, [21] it should commence early and still be a key theme of the early and in particular mid-stage of the rehabilitation process [23] to avoid physical deconditioning and ensure a more appropriate physical fitness profile to commence late-stage rehabilitation and re-conditioning.

Key elements of reconditioning entail ensuring players can physically cope with the demands of their sport and have restored the necessary physical performance profile to excel in their sport [22]. Furthermore, addressing physical limitations of the player which may have been present prior to injury or following injury (e.g., specific muscle imbalance/ poor upper body strength or cardiovascular (CV) fitness status) is important. A longer term injury often presents an opportunity to educate the patient/athlete on and get buy-in to a physical conditioning philosophy to support long term athletic development and injury minimisation. The degree of importance of physical reconditioning and the focus of the programme will in-part depend on the importance of physical fitness for the sport (e.g., the physical versus technical/tactical demands of the sport).

In football, key physical skills are developed to a high level and may be decisive in football performance whereas other physical attributes need to meet a minimum requirement to be able to cope at a certain level [19]. Top level male footballers typically cover 10–13 km during a game [79] perform about 1200 discrete bouts of activity changing every 4–6 s [116], 150–250 brief, intense actions [103], and 200–400 m of sprinting (distance covered over 7 m.s^−1^). They also perform numerous high intensity accelerations and decelerations (8-times as many accelerations as reported sprints per match), which although not resulting in speeds associated with high-intensity running are still metabolically taxing [90]. These explosive efforts involve challenging both the creatine phosphate and anaerobic glycolysis systems. Blood lactate concentrations recorded during football match play typically range from 2–12 mmol/L, with recorded individual values in excess of 12 mmol/L [66]. As such, players need to develop a very good aerobic and anaerobic cardiovascular capacity, specifically the ability to work for longer periods of time at high heart rates, to compete without the adverse effects of fatigue. The most decisive efforts leading to important outcomes/ actions are anaerobic in nature and often involve a directional change [40]. As such, restoring aerobic and anaerobic fitness, explosive acceleration, deceleration and change of direction ability as well as peak running speeds appears essential for optimal return to performance. Furthermore, there is evidence that a player’s physical conditioning (e.g., general lower body compound strength and CV fitness) is important for reducing general risk of injury. Across a range of sports, those with superior physical fitness qualities are more robust to injury [78]. Thus, returning to sport in the best physical conditioning possible is important for both performance and injury resistance (across a range of injuries, not just ACL). Recent research indicates that football players fail to fully restore their aerobic fitness (measured as VO2 max) six months following ACLR [2], indicating a greater need to prioritize and programm cardiovascular conditioning during the functional recovery period.

To ensure players restore their physical fitness profile, there is a need to adopt a return to sport/performance continuum [4, 22], incorporating a strong focus on physical fitness conditioning in the final stages, alongside an in-clinic/gym conditioning program. The return to performance continuum includes and progresses with the use of on-field rehabilitation and conditioning, return to team training, return to competitive match play and return to performance [22]. Having a specific reconditioning specialist (specific training in injury re-conditioning and RTP) involved in this process can help to bridge the gap between conventional rehabilitation (e.g., rehabilitation, physio) and the performance staff (sport science/ strength/ conditioning fitness) involved in the process. The gym-based program should ensure athletes restore their neuromuscular performance (e.g., lower limb strength, strength at adjacent joints/muscle groups for maximal strength rate of force development, power and reactive strength (where applicable)). Furthermore, we suggest incorporating where possible performance-based testing as part of RTS testing, with a view to assessing rehabilitation factors and general physical performance factors. This would ensure athletes return to sport, physically more prepared and likely place greater emphasis on this process as part of the wider functional recovery framework. RTS testing should thus involve a thorough analysis of the sport and suitable physical performance tests to ascertain the players fitness profile in relation to this needs analysis. For example, football players are traditionally tested as part of pre-season training for speed (e.g., 30 m sprint with speed gates at 5/10/20 and 30 m), change of direction ability (e.g., 505 or t-test, agility tests), aerobic and anaerobic fitness (e.g., lab based testing of running speed at lactate threshold or field based testing yo-yo/ 15–30-15 etc.), strength (e.g., squat/ mid-thigh pull/ deadlift) and power (jump height/ reactive strength index). Undertaking a physical fitness test battery as part of conventional RTS testing will support an understanding if a player is physically prepared for their sport.

## On field rehabilitation

**Fig. 1:**
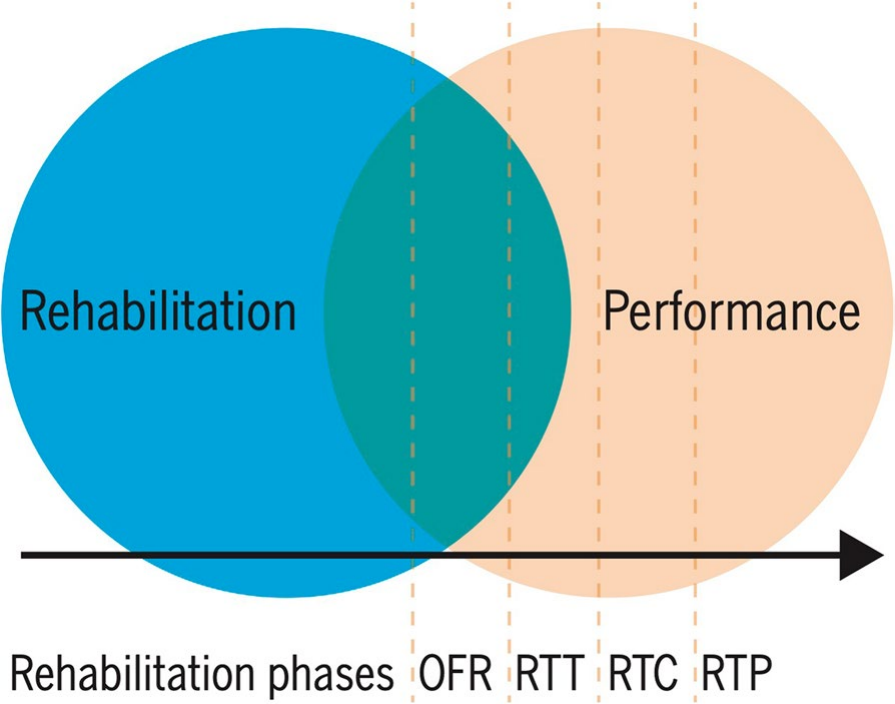
A return-to-sport process involving a gradual transition from rehabilitation to performance training and a continuum of OFR, RTT, RTC, and RTP. Abbreviations: OFR, on-field rehabilitation; RTC, return to competitive match play; RTP, return to performance; RTT, return to training (with permission [24])

In the last decade there has been an increased attention to optimize the final phase of functional recovery following ACLR. Della Villa et al. [126] introduced the concept of an On Field Rehabilitation (OFR) model to bridge the gap between rehabilitation and performance domains (Fig. 1) [22].

OFR stands between indoor rehabilitation and the return with the team. It is suggested that the patient enters this final stage only when specific objective criteria have been met, including different domains, including, but not limited to, knee extensors and knee flexors strength [23]. The OFR process has been presented in detail [24, 25]. Using the example of football OFR is a stepwise program that includes five periods of increasing functional demands for the athletes [25]. The increase of demands is both physical and cognitive to guarantee that the patient, towards the end of OFR could face some typical risk scenario for ACL injuries, that include both a mechanical and a neurocognitive perturbation. As such, the interaction with other patients/players is critically important.

Within the OFR program there are four pillars that have to be addressed:

- Movement quality (e.g., maintaining movement quality also in the unpredictable sport specific environment).
- Physical conditioning (e.g., prepare the player for the specific aerobic and anaerobic demands of football);
- Sport specific skills (e.g., recovery of individual technical and tactical skills up);
- Training load (e.g., chronically develop enough volume and high intensity metrics to justify a return to the team.

The progression of external load should be planned before every session and real time Global Positioning System (GPS) monitoring of the patient’s load during the session is suggested. The final goal of this approach is to chronically expose the athlete to the pre-injury training load (both in terms of volume metrics and high intensity metrics). This generally happens in 4–6 weeks of work with an ideal frequency of three times a week.

Technically, focusing on ACL injuries, this last part of recovery should be really focused on the injury causation. It is well established that ACL injuries happen while decelerating and thus a specific attention to training deceleration technique [80] should be implemented. Keeping the focus on the injury causation, in case of a typical football “pressing” ACL injury, the trainer should think beyond biomechanics as neurocognitive errors are really common (unpublished Gokeler A. et al. 2022) and thus visual-spatial awareness and decision-making should be trained. On the other hand, in case of a mechanical perturbation injury it is probably more important to focus on advanced perturbation training on the field to increase the functional trunk strength and patient’s capacity to absorb the typical upper body perturbations.

## Train and test athletes in a functional task environment

Recent surveys suggest that high quality evidence-based rehab is not consistently employed [35]. Rehabilitation programs mainly focus on pre-planned motor skills in a predictable environment [39]. Practicing such closed motor skills fails to comprehensively address the interaction between situational cues (sensory) and motor action responses as they relate to specific sports activities of an athlete on the field. In team ball sports, the players are immersed in a rapidly changing, unpredictable, and externally paced environment. The challenge for the player is to get to particular locations on the pitch at specific times whilst making fast action decisions, such as staying close to an opponent, in response to moment- to-moment changes [3]. These moment-to-moment changes in task and environment frame the context and demands of the sport situation in which the athlete is challenged to make appropriate movement decisions. Based on the above evidence, it appears that athletes at greatest risk of ACL injury are those who cannot cope with complex situational changes.

**Fig. 2:**
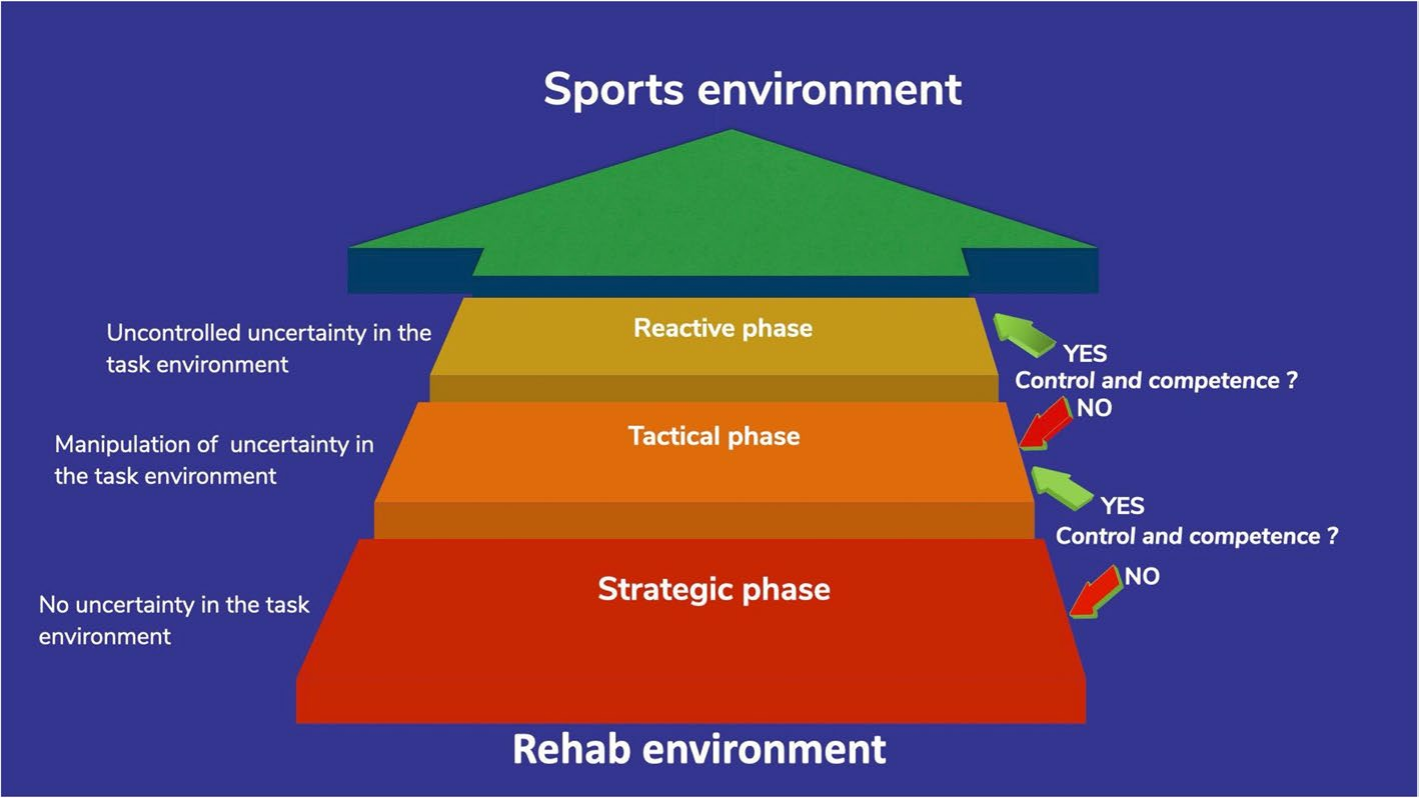
A model outlining the staged progression from controlled environments to uncontrolled uncertainty. Advancing is based on demonstration of control and movement competence [49]

Recently a framework was presented on how to organize the functional task environment during rehabilitation (Fig. 2) [49]. Clinicians can use competence and control (strategic, tactical, or reactive) as outcomes to assess whether the athlete demonstrates satisfactory performance within the functional task environment.

We propose that evaluating competence and control can manifest in various situations:

- 1. The athlete performs well from both the competence in movement strategies and perceptual-cognitive control in accomplishing goals within the functional task environment. In this case, uncertainty demands can continue to be ramped up.
- 2. The athlete demonstrates satisfactory competence in movement strategies, but with diminished sense of perceptual-cognitive control (e.g., starts to make errors) as uncertainty increases in the functional task environment. In this case, the time-dependent demands may need to be scaled down in the tactical control phase, allowing the athlete to enhance perceptual-cognitive decision-making before progressing to more uncertainty.
- 3. The athlete does not demonstrate full competence in movement strategies but maintains good perceptual-cognitive control in accomplishing goals with increased uncertainty in the functional task environment. In this case, athletes may need to spend more time in the strategic phase on specific skills in which they need to increase their movement competence (ie, more opportunity to explore the task and environmental constraints).
- 4. The athlete demonstrates both unsatisfactory competence and control in the functional task environment. In this case, the athlete may require greater focus on strategic control phase honing goal-directed attention for all rehabilitation activities due to a reduced ability to combine physical performance with perceptual-cognitive decision-making.

Increasing the complexity of functional environments with graded uncertainty may help restore both the physical and neurocognitive aspects of performance and prepare athletes for real- world sport situations [49].

## Smart technology requires smart thinking first

Researchers and clinicians have increasingly advocated the use of motion capture technology to obtain valuable quantitative measures to aid in RTS decision continuum. Currently, the 2D video-analysis of generic movements in controlled environments (e.g. squat, single- and double-leg landing) is mostly used by clinicians [38, 125, 134].

The main challenge of the next 5 years will be the definition of the proper technology for clinicians’ needs, according to the RTS stage, the sports characteristics, and the environment. Movement behavior in the laboratory does not necessarily represent movement behavior on the field because of the fundamental differences in the interactions between the patient and their environment, therefore, the on-field motion assessment in sport-specific situations has been proposed [18, 92]. Wearable sensor technology is well-suited for such mobile settings [27, 61]: for example, clinicians with direct access to a football pitch might monitor the athlete during the on-field rehabilitation and assess biomechanical asymmetries or neuromuscular risk factors.

Clinicians are aware of the great potential derived by technology in RTS. The main limitations to extensive use in daily practice are the technical skills required, the time it takes, and the interpretability of the results. The output derived is multiple (full-body kinematics, kinetics, acceleration/deceleration, muscular activation), which carries the risk of being overwhelmed by data and not being able to infer practical implications from them. Moreover, every athlete (and so every patient) moves differently and such variability further complicates the interpretation of the data. Recent works are facing these issues by proposing innovative approaches (like Principal Component Analysis, joint coordination analysis) aiming to provide a comprehensive understanding of patients’ motion [18, 34, 76]. The goal will be to cluster patients into safe or at-risk bands according to the biomechanical and neuromuscular profile. In this scenario, artificial intelligence (AI) will offer a great chance to adapt large-scale knowledge to each patient-specific RTS process. For this purpose, the establishment of shared biomechanical datasets will be a requirement for future years. Large-scale datasets are already a common practice in other fields, e.g., cancer research [101].

The wearable technology has been put through extensive biomechanical validation against marker-based motion capture, which many consider being the gold standard, and has demonstrated reliability and validity in the movement tasks commonly used in RTS [65, 92, 104]. However, it should be noted that at current there are issues regarding valid capture of frontal knee plane motion. More work in terms of improving accuracy is needed in the coming years.

The technology should not be intended as the *answer* to the RTS complexity, but as the *engine* to improve a patient’s outcomes. In 5 years, we foresee a *smarter* use of the technologies available for RTS after ACL injury. Clinicians will have a stronger technological background and the competencies to embrace the changes to the standard RTS procedures. Clinical centers will also be furnished with adequate technology according to clinicians’ needs and expertise. The RTS process will be fully technology-informed in at least one of the phases (early, middle, late). A further 5 years might be needed to achieve complete integration. Shared knowledge and datasets will be yielding the first results on technology-based sport-specific best practices for RTS. Easy-to-use software informed by AI will provide real-time clustering of patients according to their risk profile based on relevant biomechanical and neuromuscular features. A wide dataset of valuable information on the patients’ progress will be at clinicians’ disposal to improve the quality of the entire RTS process. Reports will also include patient-leveled information to promote patients’ engagement. A co-design between clinicians and technicians will be endorsed to obtain accurate and feasible real-time assessments from the earliest to the latest phases of the RTS continuum.

## RTS is more than a strength and hop test battery

The complex anatomy and function of the knee have been well recognized [5]. Understanding and explaining a complex biological system such as the knee joint is difficult and challenging. To overcome the problem of complexity, many scientists and clinicians simplify or reduce this complexity by disassembling the complex system into single units. However, the knee joint is not a simple machine put together by bones, muscles, and connective tissue. Importantly, clinicians do not treat knees, but a person who has an ACL injury. In contrast to reductionism, a complex systems theory is a field of science studying how parts of a system give rise to the collective behaviors of the system, and how the system interacts with its environment in the broadest sense [4]. A complex system approach explores the non-linear interaction between risk factors from different scales (biomechanical, neurocognitive, psychological and physiological characteristics) [2].

Sports injuries are complex phenomena, and we need a different approach [11, 13, 60]. If we acknowledge the complexity of sports injuries, we need to accept uncertainty, non-linear dynamic interactions and emergent (unpredictable) patterns. There is a course of an injury with the “expected” process of healing and return to performance. But the principle of equifinality, which means different pathways will lead to similar outcomes, is key on the RTS. So, athletes with the same injury, level, and care structure might go through a completely different journey despite reaching the same outcome. Due to the complex nature of sports injuries and the interacting factors, new patterns and not expected results can happen. The RTS process includes an individual level with athlete-related aspects (e.g., tissue healing to personal traits); an organizational level with factors including sporting club, organization and support team; and an environmental level beyond the organizational level, such as weather, playing schedule and competition level [136]. Identifying such factors, exploring the potential connections, and learning how the athlete and the system behave are essential information to top off the decision-making.

In case of an ACLR, one could argue that a part in the human machine has been replaced (ACL graft), and subsequently, the machine should function normally again. Another view could be, in analogy to a car, that patients after ACLR have rebuilt transmissions and these are not the same as the factory transmissions (native ACL) [3]. Different from cars, humans form a biological system with an inherent capability to adapt to changes. This is also where the complexity lies, as large inter-individual differences may arise as to how humans respond to these changes. Some injured athletes may indeed return to normal function and achieve their full potential and participate (sports, work) at the same level as before the injury. Others, however, may reduce their activity.

## Rehab and RTS is not only about what, but also for whom, when and how

The RTS literature is constantly looking for objective measures that allow clinicians to make appropriate decisions. This criteria-based approach aims to provide enough information to the team to make a final decision. However, such decisions will always depend on the context. An ACL injury, for instance, needs to be considered beyond the ligament or the knee or the demand of the sport. We need to look at the wholeness: an injury happens in an athlete, with his/hers individualities, participating in specific sports within a social structure in a particular place and within a specific time [16]. Decision-making based on the STAART model (Strategic Assessment of Risk and Risk tolerance) includes the importance of the contextual factors that may contribute to risk tolerance, like the pressure of media, fans, parents, coaches; the financial impact, or the importance of the game [4, 111]. Sometimes the same outcome from functional tests is interpreted differently depending on the sports modality, level, the athlete experience, potential replacement, moment of the season, etc. By the end, the contextual factors play a role not only in the decision-making but throughout the process from the acute phase to return to sports [121]. Therefore, as much as we aim to have a yes or no answer and a clear cutoff for return to sports, usually the answer is “it depends” because context matters.

The next challenge is to gather this information about the context. From a public health perspective, any health condition should consider a broader social and environmental context [45]. It is also recommended that RTS needs to be a shared decision process [4, 63]. But who are the main stakeholders, what is their potential contribution and how to include them in the process? Again, it will depend on the context. For instance, you might consider the parents for a young athlete, while an Olympic level athlete has a network with physical coaches, psychologists, doctors, and managers. To do so, it is also essential to coordinate the process through open and effective communication.

But to get to know the context, there is a need to give voice and listen to the perspectives from the main person involved in the injury rehabilitation and RTS processes [16, 17]. Most of the topics mentioned so far, like neurocognitive/neurophysiological functions, will be influenced by context. Any intervention should be tailored based not only on what we need to do but for whom, when, how and why. Engaging and empowering also required an open-minded clinician building a co-creating process for rehabilitation and RTS [46, 63]. To answer such questions, we need to include the athlete and the stakeholders actively and learn from their experiences, perspectives, and culture. By the end, they are the experts of their own context.
