# Correction: Return to sports after ACL injury 5 years from now: 10 things we must do

**DOI:** 10.1186/s40634-022-00548-x

**Published:** 2022-11-17

**Authors:** Alli Gokeler, Alberto Grassi, Roy Hoogeslag, Albert van Houten, Tim Lehman, Caroline Bolling, Matthew Buckthorpe, Grant Norte, Anne Benjaminse, Pieter Heuvelmans, Stefano Di Paolo, Igor Tak, Francesco Della Villa

**Affiliations:** 1 Centre for Orthopaedic Surgery and Sports Medicine OCON, Hengelo, The Netherlands; 2 Department of Public and Occupational Health, Amsterdam Movement Sciences, Amsterdam Collaboration On Health and Safety in Sports, Amsterdam UMC, Amsterdam, Netherlands; 3 Department Exercise and Health, Faculty of Science, Exercise Science and Neuroscience, Paderborn University, Paderborn, Germany; 4 IRCCS Istituto Ortopedico Rizzoli, Bologna, Italy; 5 Allied Health and Performance Science, St Mary’s University, Twickenham, London, England; 6 Exercise Science Program, School of Exercise and Rehabilitation Sciences, University of Toledo, Toledo, USA; 7 Center for Human Movement Sciences, University of Groningen, University Medical Center Groningen, Groningen, Netherlands; 8 School of Sport Studies, Hanze University Groningen, Groningen, the Netherlands; 9 Dipartimento Di Scienze Biomediche E Neuromotorie DIBINEM, Università Di Bologna, Bologna, BO Italy; 10 Sports Physical, Therapy Clinic Fysiotherapie Utrecht Oost, Utrecht, The Netherlands; 11 Education and Research Department, Isokinetic Medical Group, FIFA Medical Center of Excellence, Bologna, Italy

**Correction: J Exp Ortop 9, 73 (2022)**

10.1186/s40634-022-00514-7

Following publication of the original article [1], the authors identified an error in authorgroup. Tim Lehman was missing. The original article has been corrected.
